# MicroRNAs as Sensitizers of Tyrosine Kinase Inhibitor Resistance in Cancer: Small Molecule Partnerships

**DOI:** 10.3390/ph18040492

**Published:** 2025-03-28

**Authors:** Alma D. Campos-Parra, David Sánchez-Marín, Víctor Acevedo-Sánchez

**Affiliations:** 1 Instituto de Salud Pública, Universidad Veracruzana (UV), Xalapa 91190, Mexico; vicacevedo@uv.mx; 2 Posgrado en Ciencias Biológicas, Facultad de Medicina, Universidad Nacional Autónoma de México (UNAM), Ciudad de México 04360, Mexico; david_sm96@outlook.com

**Keywords:** miRNAs, TKIs, cancer, sensitizing, overcome resistance

## Abstract

Tyrosine kinase inhibitors (TKIs) have revolutionized cancer treatments by being less toxic and improving the survival of cancer patients. The greatest challenge to their success is the resistance exhibited by cancer patients. However, the potential of microRNAs (miRNAs) for sensitizing molecules to TKIs has been well recognized, with several reports publishing promising results. Nonetheless, this therapeutic window faces challenges and several often-overlooked limitations. One of the most fundamental challenges is selecting the optimal miRNA candidates for clinical trials, as miRNAs are promiscuous and regulate hundreds of targets. In this review, we describe how miRNAs enhance sensitivity to TKIs across various types of cancer. We highlight several challenges and limitations in achieving a successful collaboration between small molecules (TKIs–miRNAs). Our focus is on proposing a workflow to select the most suitable miRNA candidate, recommending several available bioinformatics tools to develop a successful therapeutic partnership between TKIs and miRNAs. We hope that this initial proposal will provide valuable support for future research.

## 1. Introduction

### 1.1. MicroRNAs

Gene modulation is essential for maintaining homeostasis and provides a key advantage by enabling cells to rapidly respond to the environment. One of the major players is microRNAs (miRNAs), which are 18~22-nucleotide RNAs capable of regulating thousands of genes at various levels of the genetic flow, thereby contributing to most cellular functions. Since their discovery in 1993 by Victor Ambros and Gary Ruvkun, over 2500 microRNAs have been identified in humans and annotated in the miRBase database [1,2,3]. Their significance in cell biology is so profound that in 2024 they were awarded the Nobel Prize in Physiology or Medicine for this merit [4].

The mechanism through which miRNAs modulate gene expression is by downregulating their target genes through base complementarity and either stalling their translation or inducing degradation through association with a protein complex called RISC (RNA-induced silencing complex) [5]. However, before miRNAs can perform their function, they are subject to several regulators such as their biogenesis, miRNAs strand selection, RNA modifications, epigenetics, transcription factors, and super enhancers. Furthermore, molecules of many types can be miRNA targets, mainly mRNAs but also long noncoding RNAs (lncRNAs), pseudogenes, and circular RNAs (circRNAs). Interestingly, a single miRNA can regulate up to 90 targets on average, and for the more versatile miRNAs, even a thousand targets, a single target can be regulated by multiple miRNAs [6,7,8]. Hence, the diversity of molecules regulated by miRNAs and the number of interactions constitutes a multilayered network of fine-tuned regulatory mechanisms and cellular functions.

Due to its relevance in cell biology, it is indisputable that loss of homeostasis in disease can alter the fine-tuned networks of interactions orchestrated by miRNAs. Several miRNAs have been studied and associated with numerous pathologies. In cancer, they can act as either oncogenes or tumor suppressors depending on the targets they have—tumor suppressor genes and oncogenes, respectively [9]. The underlying causes of their dysregulation include amplification/deletion events, altered transcription, epigenetic modifications, and defects in the miRNA biogenesis machinery [9]. Therefore, it is reasonable that by now hundreds of miRNAs have already been associated to several hallmarks of cancer such as proliferation, cell death evasion, metastasis, and angiogenesis.

In addition, like other genes, their expression can be tissue-specific, resulting in differential expression across several types of cancer. Their expression also varies depending on cancer subtype, immunological profile, stage, or cell type [10]. This is the reason why some of them have been evaluated as potential biomarkers for prognosis, diagnosis, monitoring, and evaluating treatment. To date, over 449 clinical trials for miRNAs have been performed in cancer. One of the most studied is miR-21, which has been proven to be upregulated across a wide variety of cancers and has been found to be highly expressed in body fluids in early stages. It is a promising diagnostic biomarker for multiple types of cancer, including breast, lung, colorectal, glioblastoma, and pancreatic cancer [11]. MiRNAs can be used as diagnostic markers for cancer subtypes. For instance, miR-205 helps differentiate between breast cancer subtypes, as it is upregulated in the luminal A and luminal B subtypes, less expressed in HER2-positive, and significantly downregulated in triple-negative breast cancer.

To date, no miRNA-based therapies have been approved. This is partly due to the diverse network of miRNA targets and the challenges of tissue-specific delivery methods [12,13]. As highlighted before, miRNAs form complex regulation networks with thousands of genes, so selecting a single miRNA as therapy requires strict criteria. Nonetheless, two siRNA (small interfering RNA) drugs, patisiran and givosiran, have been successful and approved by the Food and Drug Administration (FDA). Both have miRNA-like mechanisms and are used to treat polyneuropathy and acute hepatic porphyria, respectively [14,15].

The heterogeneity of the disease, on the other hand, has always been a challenge for any therapy. That is why some studies have proposed panels of biomarkers consisting of several miRNAs. In this regard, circulating miRNAs have shown promising results as biomarkers for non-invasive detection methods. For instance, Abdipourbozorgbaghi et al. 2024 proposed two panels of seven and nine circulating miRNAs for lung adenocarcinoma and squamous cell carcinoma, respectively. Additionally, four miRNAs have shown prognostic features [16]. Another example is the panel validated by MacDonagh et al. consisting of five miRNAs that are not only diagnostic biomarkers but can also help differentiate between tumors that are sensitive or resistant to cisplatin [17]. Interestingly, as mentioned in the examples above, an miRNA can be a dual biomarker. As such, the role of miRNAs in drug resistance goes beyond biomarkers. They play both an active and passive role in the mechanisms underlying drug resistance through their targets and the effectors in the downstream pathway. The processes through which drug resistance is acquired are well known; however, little is known about how miRNAs modulate these processes. They can act directly on pharmacological targets or indirectly through mechanisms such as increased drug efflux, metabolizing enzymes, cellular repair mechanisms, cell death, and proliferation/cell cycle [18].

Several miRNAs have been described with an active role in all of these processes that lead to drug resistance. For instance, Zhong et al. identified a panel of 123 dysregulated miRNAs in breast cancer with vinorelbine resistance, and 17 of them were involved in chemotherapy response signaling pathways, thus indicating miRNAs as regulators of drug resistance mechanisms [19]. Another example is the work of Zhu et al., who described a panel signature of three miRNAs in which miR-19a-3p constitutes a dual biomarker for both prognosis and drug resistance to FOLFOX [20,21].

As with chemotherapy, several miRNAs have also been described to hinder targeted therapy. For instance, miR-199b deletion has been associated with imatinib resistance indicated for Philadelphia positive chronic myeloid leukemia (CML) patients [22]. In breast cancer, miR-221 is upregulated and promotes trastuzumab resistance by targeting PTEN (phosphatase and tensin homolog) [23]. The same effect is present in breast cancer cells overexpressing the circular RNA circCDYL2, which stabilizes *GRB7* (growth factor receptor-bound protein 7), an adaptor protein involved in the signaling pathways mediated by HER2 (receptor tyrosine-protein kinase erbB-2) expression. This stabilization leads to resistance through a downstream molecule of the receptor [24].

It is worth mentioning, however, that miRNAs also contribute to drug resistance as a secondary effect of preexistent drug resistance. Alterations such as *KRAS* (GTPase KRas) mutations not only contribute to dysregulation of the miRNA biogenesis machinery but can also upregulate miRNAs such as miR-30c and miR-21 [25]. Hence, the regulation is bidirectional and, in some cases, can create feedback loops through downstream effects of their own regulation [26]. The outcome of regulation can be positive for miRNAs that are overexpressed and contribute to the tumor phenotype, or negative for miRNAs with tumor suppressor activity, whose expression is attenuated.

MiRNAs are a promising therapeutic window to overcome drug resistance through restoration of their expression. These have proven beneficial not only for inhibiting cell proliferation and invasion but also for sensitizing targeted therapy. The scope of this work is to discuss what we know about miRNAs as a potential therapy to sensitize cells to tyrosine kinase inhibitors (TKIs) and what approach should be implemented when proposing miRNAs a therapy.

### 1.2. Protein Kinases

Protein kinases are a class of enzymes that modify other molecules through phosphorylation, thus activating or deactivating their substrates. This mechanism is crucial in the physiological regulation of several processes within cells [27]. The human genome encodes for 538 protein kinases, which are enzymes that catalyze the transfer of the γ-phosphate group from ATP (adenosine triphosphate) to serine, threonine, or tyrosine residues to trigger the activation/inactivation of downstream signaling cascades that support cell proliferation, apoptosis, motility, cell cycle, differentiation, etc. [28].

Protein tyrosine kinases (PTK) are enzymes that selectively phosphorylate tyrosine residues on various substrates. At least 90 types have been described, which may be receptor tyrosine kinases (RTKs) or non-receptor tyrosine kinases (NRTKs) [29]. The RTK family encompasses a variety of transmembrane receptors that respond to growth factors, hormones, and cytokines. In humans, there are 58 known RTKs which are grouped into 20 subfamilies/classes (Table 1). RTKs are transmembrane receptors that have a similar structure consisting of an extracellular region, a transmembrane α-helix, an intracellular region that contains a juxtamembrane, a tyrosine kinase domain, and a carboxyl (C)-terminal tail [29].

RTKs are typically activated upon specific ligands binding to the extracellular region of the receptor. The interaction induces receptor dimerization or oligomerization, leading to autophosphorylation and the release of cis-autoinhibition. Once activated, RTKs recruit and phosphorylate various signaling proteins containing Src-2 homology (SH2) or phosphotyrosine-binding (PTB) domains. These domains bind to phosphotyrosine residues on the receptor and activate mediator molecules that trigger relevant cellular signaling pathways [30]. RTKs mainly activate the PI3K/protein kinase B (AKT)/mechanistic target of rapamycin (mTOR), rat sarcoma (RAS)/MAPK (mitogen-activated protein kinase), Janus kinase (JAK)/signal transducer and activator of transcription protein family (STAT), and phospholipase C (PLC)/Ca^2+^/calmodulin-dependent protein kinase-protein kinase C (CaMK-PKC) signaling pathways [31].

NRTKs are protein kinases that exhibit considerable structural variability. However, they all share a common kinase domain and typically have many other domains, such as SH2 and SH3, which are protein–protein interaction domains [32]. They are found in the cytoplasm but can also be bound to the cell membrane or be nucleus-specific. In all cases, they play a key role in signal transduction, regulating essential cellular processes such as growth, proliferation, adhesion, differentiation, migration, and apoptosis. In humans, there are 32 NRTKs, based on sequence similarity, which are classified into 10 subfamilies/classes (Table 2) [33].

Unlike RTKs, which are activated by ligand binding, activation of NRTKs involves a complex mechanism of action that employs heterologous protein–protein interaction, leading to transphosphorylation [32]. NRTKs mainly activate the JAK/STAT, FAK, and SRC signaling pathways [34].

It is well known that aberrant activation of tyrosine kinases—due to mutation, dysregulated expression and/or amplification, phosphorylation, aberrant epigenetic regulation and chromosomal translocation—is a relevant mechanism that promotes several diseases, including cancer [28]. For instance, the overactivation of RTK signaling has been associated with approximately 30% of all human cancers, such as acute myeloid leukemia, renal cell carcinoma, breast cancer, melanoma, lung adenocarcinoma, and many more [35]. In addition, NRTKs have been reported to have mutations that lead to aberrant signaling in various hematological malignancies, such as lymphomas, leukemias, and myelomas. These mutations can be either point mutations or gene fusions [34]. Tyrosine kinases have emerged as a key class for targeted therapeutics over the past two decades. Numerous small molecule-based tyrosine kinase inhibitor (TKI) therapeutics have been developed and clinically approved for several cancers. However, despite their efficacy, many of the current TKI treatments eventually lead to acquired resistance and subsequent tumor relapse.

### 1.3. Tyrosine Kinase Inhibitors (TKIs) and Mechanism of Resistance

Tyrosine kinase inhibitors (TKIs) are small molecules that compete with the adenosine triphosphate-binding site of the catalytic domain of a tyrosine kinase blocking the protein kinase signal transduction pathway [36]. TKIs are widely used as targeted therapies for multiple hematological malignancies and solid tumors, significantly improving cancer treatment. Compared to traditional chemotherapy, TKIs offer notable advantages including higher efficacy, lower toxicity, and greater specificity [29].

In 2001, imatinib became the first TKI to be approved by the FDA for the treatment of CML. Since then, nearly 50 TKIs have received FDA approval, and they inhibit key proteins such as EGFR, ALK, ROS1, HER2, VEGFR, RET, MET, MEK, FGFR, PDGFR, NTRK, and KIT, among others [37]. Despite their initial efficacy, TKIs are limited by the development of acquired resistance. In this work, we focus only on TKIs for which miRNAs have been reported to overcome resistance and restore their therapeutic effect.

Imatinib is a TKI directed against the BCR-ABL oncoprotein by preventing BCR-ABL autophosphorylation and blocking downstream signaling pathways such as Ras/Raf/MAPK, PI3K/AKT/mTOR, and JAK/STAT. It also inhibits tyrosine kinases such as KIT and PDGFRα [38]. Imatinib has become the first-line treatment for newly diagnosed CML due to its excellent response in patients with chronic phase CML [38]. However, the main obstacle to its success is the development of therapeutic resistance characterized mainly by the presence of point mutations (>100 described) within the ABL tyrosine kinase of the BCR-ABL oncoprotein as well as amplification of the BCR-ABL1 oncogene [39]. Some mutations that confer partial resistance to imatinib can be overcome with higher doses of the drug. However, most cases require a switch to a second- or third-generation TKI [39]. Specifically, Y253H, E255K, and F359C mutations confer resistance to imatinib and the second-generation TKI, nilotinib. Additionally, V299L and F317L mutations confer resistance to both imatinib and the second-generation TKI dasatinib [40]. In addition, the T315I mutation is associated with resistance to all currently available TKIs except ponatinib, which is a third-generation TKI [41].

Imatinib is also used as a treatment for unresectable and metastatic gastrointestinal stromal tumor (GIST), significantly improving survival and delaying disease progression in many patients [42]. Those patients harboring mutations in exon 11 typically respond well to imatinib, but *PDGFRA* D842V mutations confer resistance. Evaluating the mutation spectrum in these patients is essential, as resistance can develop after 6 months due to the presence of *PDGFRA* D842V, and *KIT* exon 9 mutations [42].

The first-generation reversible EGFR-TKIs, gefitinib and erlotinib, have shown benefits in non-small cell lung cancer (NSCLC) patients carrying EGFR-sensitizing mutations (L858R in exon 21 and exon 19 deletions) [43]. The main mechanism of resistance for gefitinib and erlotinib is the presence of the *EGFR*-T790M mutation [43]. *HER2* mutations, *MET* amplifications, or histological changes to small cell lung cancer are secondary mechanisms of resistance to erlotinib and gefitinib [44]. Third-generation EGFR-TKIs, such as osimertinib, inhibit the T790M mutation, which is resistant to gefitinib and erlotinib. However, it has also been found to be efficient in inhibiting EGFR-sensitizing mutations, being superior to first-generation EGFR-TKIs. It even has efficacy in patients with metastases in the central nervous system (CNS) [38]. However, as the disease progresses, *EGFR* mutations C797S and G742S have been reported to be resistant to osimertinib. Other resistance mechanisms included *MET* amplification, *HER2* amplification, *PIK3CA* (phosphoinositide 3-kinase alpha), *KRAS*, *BRAF* (serine/threonine-protein kinase B-raf), or a rare *EGFR* secondary mutation [44].

Sorafenib is another TKI that was approved in 2007 for the treatment of advanced liver cancer. It was later approved as the first-line therapy for hepatocellular carcinoma (HCC), with limited success for patients (30–35% of patients respond to this TKI). Furthermore, acquired resistance often develops within 6 months [45]. Sorafenib is a multikinase inhibitor since it inhibits Raf-1 (RAF proto-oncogene serine/threonine-protein kinase), VEGFR-1, VEGFR-2, VEGFR-3, PDGFR-β, c-KIT, FLT-3, and RET with antiangiogenic, antiproliferative, and proapoptotic properties [46]. The precise mechanism of resistance to sorafenib is unclear, as there are several mechanisms that contribute to this resistance, such as high EGFR and c-Jun expression, autophagy, hypoxia, epithelial-mesenchymal transition (EMT), tumor microenvironment, epigenetic regulation, ferroptosis, PI3K/Akt signaling pathway, Hippo/Yap pathway, and IL-6/STAT3 signaling, among others [47,48].

Fexagratinib (AZD4547) is a potent reversible TKI of FGFR-1, 2, and 3 receptors [49]. The TARGET (technology-enhanced acceleration of germline evaluation for therapy) study (phase I/II multicenter trial), recently published, reported a limited benefit of fexagratinib in patients with recurrent *FGFR-TACC* gene fusion diffuse high-grade gliomas [50]. In the Lung-MAP substudy of previously treated patients with stage IV squamous lung cancer and FGFR activation, fexagratinib showed acceptable tolerability but poor efficacy [51]. The same occurred in the NCI-MATCH (National Cancer Institute-molecular analysis for therapy choice) trial of tumors such as breast, urothelial, and cervical tumors with *FGFR1-3* aberrations, as fexagratinib showed modest benefit only in patients with FGFR1-3 point mutations or fusions [52]. In luminal subtype metastatic breast cancer, fexagratinib demonstrated clinical benefit in combination with anastrozole or letrozole, particularly in patients who progressed on hormonal therapy. This benefit was observed independently of *FGFR* amplification but was especially noted in those whose breast cancers showed a gene expression pattern linked to FGF action [53]. To date, mechanisms of resistance to fexagratinib remain unreported, likely due to its limited efficacy.

Dasatinib is a second-generation small-molecule used to treat patients with CML that is chronic, blastic, or accompanied by accelerated phase who are resistant or intolerant to previous treatments [54]. This TKI is a multitarget kinase inhibitor. It inhibits BCR-ABL, Src kinases (Fyn, Yes, Src, and Lyk) and c-Kit [55]. Dasatinib inhibits both wild-type and most mutant BCR-ABL kinase activity, except for the T315I-resistant mutation [56]. F317L is another mutation that has been detected in dasatinib-resistant patients [57].

Cabozantinib is an inhibitor of multiple tyrosine kinase receptors involved in tumor growth and angiogenesis, such as MET, AXL, and VEGF receptors. It also inhibits other tyrosine kinases including GAS 6 (growth arrest-specific protein 6), receptor AXL, RET, ROS1, TYRO3, MER, KIT, FLT3, and TIE2 (angiopoietin-1 receptor) [58]. Cabozantinib is approved in Europe and the United States for the treatment of advanced renal cell carcinoma (RCC), HCC in patients previously treated with sorafenib, and unresectable locally advanced or metastatic medullary thyroid cancer [58]. Some of the mechanisms of resistance to cabozantinib have been shown to involve the secretion of proangiogenic factors [59]. *MDM2* (E3 ubiquitin-protein ligase Mdm2) amplification has been identified as resistance mechanism in patients with RET-a rearranged lung cancers [60].

Given the above, it is interesting to note that cancer remains relentless, as it develops different mechanisms responsible for the acquisition of resistance to several TKIs. Therefore, knowledge of these mechanisms has served to potentially study, propose, and use biomarkers of resistance in order to predict the efficacy of these drugs or, alternatively, that could serve as targets to overcome resistance in cancer patients. This is where small molecules like miRNAs come into play, as they can be used as sensitizers of TKIs. While this approach is novel, it also presents significant challenges in clinical practice, as we will explore below.

## 2. MiRNAs and TKIs: Association of Small Molecules in Overcoming Resistance

### 2.1. MiRNAs Sensitizing to Imatinib

**Figure 1:**
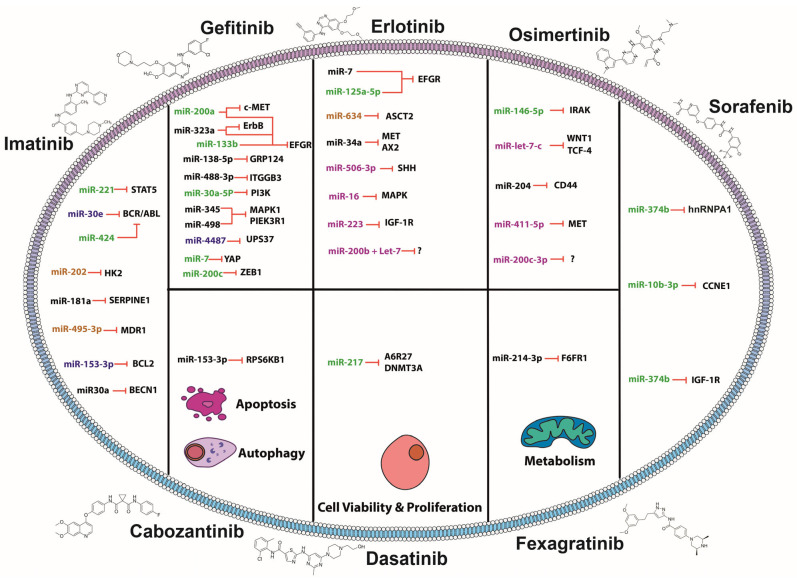
Compilation of identified miRNAs as sensitizers of TKIs and their described implications in treatment rescue: induction of apoptosis (miRNAs in green), autophagy (miRNAs in purple), metabolism (miRNAs in orange), cell viability and proliferation (miRNAs in pink).

Since the first report of imatinib resistance, in the early 2000s, great effort has been expended describing the mutations that drive the resistance and by which mechanisms it is acquired [61]. Also described along the way have been several ways to face this challenge, mainly, by second- and third-generation inhibitors, but also by describing molecules that can modulate therapy efficacy such as miRNAs. These mechanisms vary widely depending on the miRNA targets and their biological fun—signaling transduction, apoptosis, metabolism, and autophagy—and when the miRNAs expression is restored, these mechanisms are inhibited, featuring miRNAs’ potential therapeutic opportunity (Figure 1). For instance, miR-221 was found to be significantly downregulated in CML non-responsive patients. In vitro assays demonstrated that miR-221 sensitizes CML cells to imatinib. Moreover, *STAT5* was identified as an miR-221 target through an array of apoptosis-related genes, and its gain of function and *STAT5* knockdown led to sensitization to treatment in CML cell lines (K562 and imatinib-resistant K562/G). This is due to *STAT5*’s well-known oncogene roles in CML through cell cycle, apoptosis, ROS (reactive oxygen species) generation and drug resistance. In fact, its activation can induce *BCR-ABL* expression, a driver gene for several types of leukemia and a target of imatinib [62]. In this sense, coadjuvant therapy is essential for this gene, and miRNAs as sensitizers complementing TKIs therapy is really valuable, as the target is not only at the protein level but also post transcriptional, reducing the proteins pool available [63]. Another case is miR-424, which is also downregulated, and upon its restoration, the BCR-ABL treatment resistant cell line—K562—stops proliferating, and apoptosis is induced [64]. On the other hand, it has been identified that CML cells resistant to imatinib exhibit upregulated glycolysis due to increased glucose uptake, lactate production, and upregulated glycolysis enzymes [65]. Remarkably, it has been described that miR-202 can not only revert cells to a less glucose-dependent phenotype by targeting Hexokinase 2 but also sensitize cells to imatinib (imatinib resistant cell line originating from K562 parental CML cell lines) [66]. One of the most common mechanisms of drug resistance is the efflux of drugs through pumps such as the multidrug resistance 1 (*MDR1*) gene. Remarkably, these genes are subject of miRNAs regulation. In CML, miR-495-3p is notably downregulated, and its predicted targets upregulate, such as *MDR1*. Its overexpression in imatinib-resistant T315I-mutant cells, rescued the sensitivity to imatinib through hindering the drug efflux [67].

Autophagy is well known to have a dual and controversial role in cancer, as it can be a tumor suppressor or an oncogenic process [68]. Interestingly, a mechanism has been described for imatinib resistance through both inhibition of the autophagy factor and a pro-autophagy factor in CML and GIST, respectively. On one hand, it has been described that miR-153-3p can sensitize imatinib-resistant CML cells to imatinib by targeting *Bcl-2*, which inhibits autophagy by binding to Beclin1 [69]. On the other hand, miR-30a can sensitize GIST cells (GIST-T1 and GIST-882 cells resistant to imatinib) to imatinib by targeting Beclin1, which is involved in autophagosomes formation [70]. These last two studies show a scenario that represents two mechanisms of acquired resistance to imatinib through the inhibition/promotion of the same process (autophagy), reinforcing the importance of miRNAs in these mechanisms and their relevance as a potential therapy.

### 2.2. MiRNAs Sensitizing to Gefitinib

A prime example of an miRNA that sensitizes NSCLC cell lines that are resistant to gefitinib is miR-200a. This miRNA targets both *EGFR* and c-*Met*, two tyrosine kinases that have induced resistance to gefitinib while promoting metastasis through shared effector pathways in gefitinib-resistant NSCLC cell lines [71]. Another example is miR-323a-3p, which can target two upregulated tyrosine kinases in gefitinib-resistant colorectal cancer cells (HCT116 GR (gefitinib-resistant GR), LoVo GR, SW620 GR, and HT-29 GR cells)—*ErbB* and *EGFR*—representing a potential multi-treatment opportunity to overcome gefitinib resistance and induce apoptosis [72]. On the other hand, there are miRNAs that can have a single target that has a key role. For instance, a TKR itself. This is the case for miR-133b, which targets *EGFR* and not only sensitizes cells to gefitinib, but can modulate apoptosis, proliferation, and invasion as well, thus creating another potential target to overcome drug resistance. This conclusion was made after observing a significantly higher sensitivity to gefitinib in H1650, H1975, and PC-9 cells transfected with miR-133b mimic [73]. AXL is another TKR that promotes pathway activation through stabilization of β-Catenin; however, after its knock-down, several miRNAs are downregulated, miR-347a being the most downregulated and miR-548b being the most upregulated [74]. In addition, it has been described that by inhibiting miR-347a, it could target *Wnt5a* (protein Wnt-5a) and reduce tumors in gefitinib-resistant NSCLC cells in xenograft (gefitinib-resistant HCC827 cell line transplanted into xenograft). Moreover, miR-548b, when upregulated, could target *CCNB1* (G2/mitotic-specific cyclin-B1), contributing to cell cycle regulation and reversing the tumor phenotype [75]. Another miRNA that targets *EGFR* is miR-4487. A synergistic effect of gefitinib plus miR-4487 has been reported, as this miRNA mediates *EGFR* degradation by autophagy in the gefitinib-resistant cell sublines PC-9/GR and HCC827/GR, constituting a potential therapeutic target for EGFR variants that leads to its endocytosis [76].

Another miRNA involved in gefitinib sensitivity is miR-138-5p, which targets *GPR124* (adhesion G protein-coupled receptor A2) and whose expression is inversely correlated in NSCLC specimens. Furthermore, miR-138-5p restoration can sensitize gefitinib-resistant cells (PC9GR cell line resistant model generated by continually exposing PC9 NSCLC cells to gefitinib for six months) [77]. Receptors are not the only proteins causing potential drug resistance, but other cell-membrane proteins can contribute as well. Integrin β3, a cell adhesion and signaling mediator protein, has been associated to drug resistance in various cancer types [78,79]. In an NSCLC model (NSCLC HCC827, H1975, A549, H292, and H1299 gefitinib-resistant cell lines), integrin β3 constitutes a target of miR-483-3p that, when overexpressing the miRNA, causes the inhibition of the FAK/Erk pathway and suppression of the EMT and metastatic phenotype [80]. Interestingly, another study using in vitro and in vivo models resistant to gefitinib (H1650 with acquired gefitinib-resistance—H1650GR—and H1650GR cells implanted in a mouse model), demonstrated that miR-30a-5p sensitizes these models to gefitinib, inducing apoptosis and hindering cell invasion and migrations, as it is a direct target. Therefore, the combination of gefitinib plus the miRNA represents an opportunity to overcome drug resistance [81].

As previously mentioned, the restoration of miRNAs that confer sensitivity to gefitinib seems to be important. In this regard, it has been described that a derivative of resveratrol—trans-3,5,4-trimethoxystilbene (TMS)—can effectively restore miR-345 and miR-498 expression and sensitize lung cancer cells to gefitinib (PC-9/gefitinib resistant) through MAPK1 and PIK3R1 (phosphatidylinositol 3-kinase regulatory subunit alpha) and ultimately regulate the MAPK/c-Fos and AKT/Bcl-2 (apoptosis regulator Bcl-2) signaling pathways [82].

Negative regulators of the pathways also confer resistance to TKIs. For example, miR-214 negatively regulates *PTEN*, thus activating the PI3K/AKT pathway. Therefore, deletion of miR-214-sensitized *EGFR*-mutant and gefitinib-resistant HCC827 cells to the drug [83].

Hippo signaling is another pathway associated with gefitinib resistance. Interestingly, miR-7 was found to be downregulated in gefitinib resistance cell lines (gefitinib resistance H1975 cells) compared to those that respond to treatment (gefitinib sensitivity PC9 cells). It was then described that miR-7 can target *YAP* (yes-associated protein 1), promoting apoptosis and sensitizing cells to gefitinib. However, what is most noteworthy is the significantly higher expression of miR-7 in the serum of healthy patients compared to that of NSCLC patients. Even more interestingly is the fact that exosomes containing miR-7 could transfer it from gefitinib-resistant cells to gefitinib-sensitive cells. This, in turn, suggests a dual potential role for miR-7 as both a biomarker and a therapeutic target [84]. At the transcriptional level, miRNAs also have a wide range of targets that can impact TKI resistance. For instance, miR-200c downregulated in NSCLC can restore sensitization to gefitinib in the resistant cell line PC-9-ZD through targeting *ZEB1* (zinc finger E-box-binding homeobox 1), and then, by inhibiting the PI3K/Akt signaling pathway and inducing apoptosis [85].

Although efforts have been made to better understand the role of miRNAs in sensitizing gefitinib-resistant cells, the mechanism of some of them remains unknown. For example, miR-200a and miR-200c were described to be up-regulated in extracellular vesicles found in the plasma of good responders to gefitinib. Furthermore, in vitro experiments in EGFR wild-type cells (CL1-5) demonstrated that miR-200c overexpression led to downregulation of the EGFR pathway, increased sensitivity to gefitinib, along with induction of proapoptotic factors [86].

There are also miRNAs that can impact the tumoral phenotype directly by key targets. For example, miR-634, targets *ASCT2* (neutral amino acid transporter B0), a glutamine transporter, thus reducing glutaminolysis, a crucial pathway for cancer cells to obtain nutrients. Interestingly, miR-634 as a topical treatment for cutaneous squamous cell carcinoma (cSCC) was shown to improve the efficacy of gefitinib [87].

### 2.3. MiRNAs Sensitizing to Erlotinib

Erlotinib is a first-generation TKI targeting EGFR, and miR-7 is a miRNA that acts as adjuvant with erlotinib in head and neck cancer. Kalinowsky et al. observed that in HN5 (erlotinib-sensitive), SCC-25 (intermediate sensitivity), and FaDu (erlotinib-resistant) head and neck cancer cells, miR-7 binds to EGFR mRNA, decreasing the levels of p-EGFR and p-Akt. This miRNA reduces H5 cell viability, and in a xenograft mouse model, decreases tumor volume and Akt phosphorylation. Combining miR-7 with erlotinib produces synergy, resulting in decreased viability of erlotinib-resistant cells and decreased p-EGFR and p-Akt in these cells [88]. On the other hand, in cutaneous squamous cell carcinoma (A431 cells), miR-634 inhibits the growth of these cells by inducing their apoptosis through cleaved caspase-3 and PARP (poly [ADP-ribose] polymerase), and by causing mitochondrial damage. This miRNA also decreases the levels of various proteins such as LAMP2 (lysosome-associated membrane glycoprotein 2), NRF2 (nuclear factor erythroid 2-related factor 2), BIRC5 (baculoviral IAP repeat-containing protein 5), OPA1 (optic atrophy protein 1), XIAP (X-linked inhibitor of apoptosis protein), APIP (APAF1-interacting protein), and TFAM (transcription factor A, mitochondrial). An ionic liquid transdermal system (ILTS)-based ointment containing miR-634 inhibits tumor growth in A431 cell xenograft mice in combination with erlotinib or gefitinib. Binding of miR-634 to *ASCT2* mRNA causes energetic stress by reducing lactate, glutamine, ATP, and the GSH (glutathione)/GSSG (glutathione disulfide) ratio levels and increases ROS production, sensitizing tumor cells to both TKIs [87]. In HCC, the erlotinib–miR-34a combination produces synergy and decreases the proliferation of these tumor cells (HepG2, Huh7, Hep3B, and C3A). This treatment has the same synergistic effect on the proliferation of erlotinib-resistant NSCLC cells (A549, H460, H1299, H226, HCC827res) so that miR-34a restores sensitivity to this TKI. However, the mechanism of this effect is unknown, but could be mediated by inhibition of *MET* or *AXL* by miR-34a [89]. Other miRNAs have been described as restoring the sensitivity of NSCLC cells to erlotinib. For example, in erlotinib-resistant HCC4006^ER4^ cells, miR-506-3p binds to *SHH* (Sonic hedgehog protein) mRNA and inhibits its expression, altering the EMT in the cells by decreasing vimentin and N-cadherin levels, while increasing E-cadherin levels. It also decreases the migration capacity and pluripotency of the cells by reducing the expression levels of cancer stem cell (CSC) markers such as SOX2 (transcription factor SOX-2), NANOG (homeobox protein NANOG), ALDH1A1 (aldehyde dehydrogenase 1A1), POU5F1 (POU domain, class 5, transcription factor 1), KLF4 (Krueppel-like factor 4), and CD44. Overexpression of miR-506-3p and administration of erlotinib induces apoptosis of HCC4006^ER4^ cells, and thereby, increases sensitivity to erlotinib. Furthermore, this combination reduces cell migration and colony-forming capacity, as well as the formation of smaller spheroids. All these effects were reversed when the recombinant SHH protein was administered, demonstrating the role of miR-506-3p/SHH in these processes [90].

Treatment with erlotinib and/or selumetinib plus miR-16 decreases the viability and proliferation of erlotinib-resistant A549 and NCI-H2009 cells (with activating KRAS G12S and G12A mutations) through a mechanism mediated by miR-16 binding to *MAPK3* and *MAP2K1* mRNA. Moreover, migration of these cells was inhibited after miR-16–erlotinib and miR-16–erlotinib–selumetinib treatments. In a murine xenograft model with A549 cells, both treatments decrease tumor volume, demonstrating that miR-16 sensitizes NSCLC cells to erlotinib or selumetinib [91]. In erlotinib-resistant NSCLC cells (PC-9/ER), miR-223 binds to *IGF-1R* and affects the IGF-1R/PI3K/Akt/mTOR signaling pathway by reducing the phosphorylation levels of IGF-1R, Akt, S6, and P70S6K (ribosomal protein S6), decreasing cell viability and inducing cell apoptosis after erlotinib treatment. On the other hand, in PC-9/ER CD133+ cells with CSC characteristics, miR-223 transfection and erlotinib treatment decreased the viability of these cells. Moreover, in a PC-9/ER xenograft mouse model, tumors with high levels of miR-223 had lower weight/volume and mice had increased survival [92,93]. Other miRNAs promote sensitization of NSCLC cells to erlotinib, even though their target gene is unknown. For example, miR-125a-5p reduces EGFR mRNA expression in A549 cells; however, it is unknown whether this is through direct binding. Both miR-125a and erlotinib alone reduce survival and increase apoptosis of these cells; however, the erlotinib-miR-125a combination acts synergistically and further enhances these effects [94]. A549 NSCLC cells treated with TGF-β1 generate cells with mesenchymal phenotype (A549M), which express CSC markers such as SOX2, NANOG, EpCAM (epithelial cell adhesion molecule), and are resistant to erlotinib. MiR-200 (a,b,c) and Let-7 (b,c) inhibit the proliferation of these cells and sensitize them to erlotinib treatment. However, the miR-200b + let-7c combination shows greater effect in inhibiting proliferation and reduces *ZEB1* expression while increasing E-cadherin levels, reversing EMT [95]. On the other hand, the combination of miR-34a with various TKIs such as erlotinib, afatinib, rociletinib, and osimertinib produces synergy and inhibits proliferation of cells with primary resistance (H460, A549, H226, and H1299; EGFR-wild type), secondary resistance (H1975, EGFR-L858R/T790M), and with acquired resistance (HCC827res) to erlotinib [96]. The above demonstrates the contribution of miRNAs to the effect of erlotinib in counteracting resistance to this TKI.

### 2.4. MiRNAs Sensitizing to Osimertinib

In NSCLC, several miRNAs have been described as increasing the sensitivity of resistant tumor cells to osimertinib treatment. For instance, miR-146b-5p promotes apoptosis of PE2988 and PE3479 NSCLC cells (isolated from malignant pleural effusions with exonic *EGFR* mutations and deletions) in the presence of osimertinib, favoring cleavage of caspase-3 and PARP by miR-146b-5p binding to *IRAK* mRNA. The miR-146b-5p/*IRAK* (Interleukin-1 receptor-associated kinase 1) interaction also suppresses NF-κB, inhibiting the production of IL-6 (interleukin-6) and IL-8 (interleukin-8), which contribute to osimertinib resistance in this type of cancer [97]. MiR-let-7c sensitizes H1975 (EGFR L858R/T790M double mutation) and HCC827-T790M NSCLC cells (T790M mutation acquired by continuous exposure to erlotinib and MET inhibitor) to osimertinib by decreasing tumor cell proliferation and invasion in addition to reversing EMT through increased E-cadherin and decreased ZEB1. *WNT1* (Proto-oncogene Wnt-1) and *TCF-4* (T-cell factor 4) transcripts are targets of miR-let-7c and are epigenetically inhibited through increased methylation of their promoter regions. Silencing of both genes also increased the cytotoxic effects of osimertinib [98]. MiR-204 inhibits *CD44* (cancer stem cell marker) in HCC827/gef and H1975/AZD18 NSCLC cells (resistant cells to osimertinib by continuous exposure to this TKI), repressing the pluripotency of these cells by reducing spheroid formation and promoting osimertinib-induced apoptosis. In 2D cultures, besides *CD44* inhibition, there was a decrease in N-cadherin, SOX2, snail and vimentin proteins, and an increase in E-cadherin. MiR-204 decreased viability, inhibited migration and invasion, and induced apoptosis in these cells after activation of caspase-9 and increased BimEL (Bcl2-interacting mediator of cell death) levels, thereby sensitizing them to osimertinib treatment. These results were validated in vivo using a xenograft mouse model (H1975/AZD18 cells). Administration of osimertinib to mice reduced tumor volume in those with high levels of miR-204, leading to a lower number of Ki67+ (proliferation marker protein Ki-67) cells and increased apoptosis in these xenograft cells [99]. Meanwhile, miR-411-5p edited at position 5 of the seed region (inosine replaced by guanosine) binds to *MET* inhibiting ERK and Elk phosphorylation (EPH-like kinase 6) and decreasing expression of c-Fos, c-Jun (transcription factor Jun) and c-Myc (proto-oncogene c-Myc), thereby altering the ERK/MAPK and PI3K/AKT signaling pathways and contributing to TKI resistance. In combination with osimertinib, miR-411-5p reduced proliferation and increased apoptosis in HCC827R and PC9R lung cancer cells [100]. Additionally, another study reported that miR-200c-3p inhibits EMT in H1975/AZD lung cancer cells (cells resistant to osimertinib generated by gradually increasing the concentration of this TKI) by reducing the expression of N-cadherin and ZEB1 proteins and increasing the expression of E-cadherin. Moreover, miR-200c-3p also inhibits migration and promotes apoptosis of lung cancer cells by increasing caspase-3 and PARP cleavage in combination with osimertinib [101]. These studies highlight the contribution of miRNAs to the effect of osimertinib in counteracting resistance to this TKI.

### 2.5. MiRNAs Sensitizing to Sorafenib

In hepatocellular carcinoma, several miRNAs have been reported to sensitize these cells to sorafenib treatment. For example, in Hep3B-R and HCCLM3-R cells (both resistant by exposure to increasing concentrations of sorafenib), miR-374b binds to *hnRNPA1* (heterogeneous nuclear ribonucleoprotein A1) mRNA, thereby reducing *PKM2* (pyruvate kinase PKM) levels. The decrease in PKM2 is related to the inhibition of glycolysis, as there is lower ATP and lactate production as well as less glucose uptake in Hep3B-R and HCCLM3-R cells. In addition, *PKM2* silencing decreases the levels of GLUT1 (glucose transporter type 1), HK-I (Hexokinase-1), c-Myc, HIF-1α (hypoxia-inducible factor 1-alpha), and STAT3. The combination of miR-374b with sorafenib decreases the survival of sorafenib-resistant cells by increasing their apoptosis. This combination also decreases the colony-forming capacity of these cells, while in a xenograft mouse model (Hep3B-R), it inhibits tumor growth as well as hnRNPA1 and PKM2 expression [102]. Meanwhile, overexpression of miR-10b-3p decreases the levels of its target, *CCNE1* (G1/S-specific cyclin-E1) and decreases Rb (retinoblastoma) protein in Huh-7R and HepG2R cells (resistant due to continuous sorafenib treatment). MiR-10b-3p promotes apoptosis in these cells after sorafenib treatment through increased cleaved caspase-3 and PARP-1. Furthermore, sorafenib induces miR-10-3p expression in these cells [103]. Another study reported that combined miR-122 and sorafenib treatment decreases viability and increases apoptosis in Huh7-DR3, PLC-DR3 cells (resistant due to chronic exposure to sorafenib) through a mechanism mediated by miR-122 binding to its target *IGF-1R*. Inhibition of IGF-1R (using IGF-1R inhibitors NVP-AEW541 or PPP [picropodophyllin]) combined with sorafenib increases apoptosis in hepatocellular carcinoma cells, demonstrating the role of IGF-1R in sorafenib resistance. These sorafenib-resistant cells have high levels of p-IGF-1R, p-AKT1, p-ERK1/2, p-STAT3, and RAF1, but IGF-1R inhibition reduces RAF1 and p-ERK1/2 levels as well as decreasing colony formation and viability after sorafenib treatment. Analysis of miR-122 and *IGF-1R* expression in tissue samples from HCC patients shows that miR-122 is found at low levels in patients resistant to sorafenib treatment compared to those patients sensitive to this TKI, while *IGF-1R* levels are high in sorafenib-resistant patients [104].

### 2.6. MiRNAs Sensitizing to Fexagratinib

AZD4547, also known as fexagratinib, is an FGFR 1-4 inhibitor. MiR-214-3p inhibits proliferation, migration, and invasion by targeting *FGFR1*. However, in NSCLC overexpressing FGFR1, miR-214-3p expression is notably downregulated. In this regard, Yang et al., reported that co-treatment with miR-214-3p and AZD4547 has synergistic antitumor effects in in vitro (H1581 NSCLC cells) and in vivo models (orthotopic lung cancer mouse models were established using H1581). These findings provide new insights into the prognosis and treatment of patients with *FGFR1*-amplified lung cancer [105].

### 2.7. MiRNAs Sensitizing to Dasatinib

In CML, miR-217 sensitizes dasatinib-resistant cells through binding to several targets, including DNMT3A (DNA (cytosine-5)-methyltransferase 3A) and AGR2 (anterior gradient protein 2 homolog). In K562DR cells (resistant by exposure to increasing concentrations of dasatinib for 1 year), miR-217 overexpression and subsequent treatment with dasatinib reduces the viability of these cells by mir-217 targeting DNMT3A. This effect was similarly observed upon silencing of DNMT3A with a siRNA, confirming its role [106]. MiR-217 also downregulates AGR2 in K562DR cells and, in combination with dasatinib, increases the apoptosis of these cells. In a xenograft model with K562DR cells overexpressing miR-217, dasatinib co-treatment increased mice survival compared to those with low levels of this miRNA. AGR2 silencing in K562DR cells and in the xenograft showed the same results, demonstrating that miR-217 sensitizes cells resistant to dasatinib [107].

### 2.8. MiRNAs Sensitizing to Cabozantinib

RET mutations are common in medullary thyroid cancer, and the primary treatment is a TKI that blocks the activity of this receptor, with cabozantinib being the most widely used, however, without promising results. In medullary thyroid cancer xenografts, the combined effect of miR-153-3p, which targets RET, and cabozantinib was tested. The results showed tumor growth inhibition and reversal of cabozantinib resistance. Interestingly, the researchers employed systemic miRNA delivery using EDV™ nanocells [108].

The role of miRNAs in TKIs therapy highlights the impact they have in sensitizing targeted therapy while suppressing the tumoral phenotype. A key challenge is the diversity of miRNAs’ specific profiles across tissues, which creates a dilemma: While miRNAs represent potential therapeutic targets, their redundancy in regulating shared targets may limit their specificity. This redundancy can be both an advantage, if there is a coordinated downregulation of key genes, but also a disadvantage, if there are genes with important physiological functions. This is why it is important to discuss the challenges that miRNAs face as therapeutic molecules but, more importantly, is to define the pathway toward their clinical implementation.

## 3. Methodological and Clinical Challenges for the Use of miRNAs as Sensitizers of TKIs

Several miRNAs have been studied as potential therapeutic targets; however, most have not made it to clinical trial yet due to the several challenges they face in accomplishing therapeutic efficacy. One of the major problems, similar to other RNA-based therapies, is the delivery method. Two approaches have been explored: (1) miRNA mimics, which are synthetic sequences that restore the function of tumor suppressor miRNAs, and (2) antimiRNAs, which have the opposite effect, and its goal is to silence oncomiRNAs. Although these molecules have been proven to be efficient at either restoring the miRNA function or suppressing it, in the case of mimics, their effect can be limited to the competition for the protein machinery on which miRNAs rely to knockdown their target genes. This competition may arise from other RNAs that share the same sites for binding their targets or with others. In regard to antimiRNAs, the main challenge these molecules face is the opposite effect, in which other molecules can act as sponges compromising their function, for instance long non-coding RNAs [109]. Remarkably, endogenous sponges are an interesting target of study not only because they represent an upstream mechanism to regulate miRNAs functions—acting as a double check point in a three-axis strategy of gene modulation—but also because the mechanism principle is used to design synthetic RNAs that work as sponges [110]. Specifically, circular RNAs are major sponges of miRNAs and their aberrant expression can lead to several downstream alterations in miRNA-regulated pathways [111]. Notably, a tool to design microRNA sponge sequences has been developed; miRsong [112]. In any case, using mimics or antimiRNAs as therapeutics, its administration implicates the consideration of three factors: (1) delivery method, (2) molecule stability, and (3) immunological response and cytotoxicity.

Several molecules have been explored as potential delivery methods, which can be administered either locally or systemically. Local treatments have primarily been proposed to treat solid tumors. For instance, Inoue et al. developed a treatment for cutaneous squamous cell carcinoma with miR-634 as an ointment that improved the co-treatment with EGFR-based therapy [87]. In contrast, metastatic tumors and hematological cancer require different approaches. Regardless of the vehicle for delivery, as with other RNA molecules-based therapies, microRNAs therapeutics face challenges related to stability within the organism and the cell. Several chemical modifications are incorporated into the sequences in order to guarantee their stability, and the choice depends on the molecule type, their target, and their localization. For example, antisense oligonucleotides with fluoro, amino, or methyl group on the 2′-OH ribose are used for nuclease degradation evasion and enhanced stability [113]. Another example are locked nucleic acid (LNA) bases, which enhance microRNA binding affinity to the LNA antisense [114]. Regarding the vehicles, several options have been explored ranging from systems such as viral vectors, to non-biological approaches such as lipid-based material, nanoparticles, and vesicles. However, this work will not delve into their differences. What is worth mentioning is that their differences consist on the immunological response they might trigger, the efficiency of the delivery, and target specificity [115].

Even though these are critical factors to be considered, less is discussed about the criteria to select and propose miRNAs as therapy. As mentioned before, a single miRNA has from dozens to even thousands of potential targets, and not only mRNAs but several other molecules such as lncRNAs, proteins, and other non-canonical targets. Therefore, we must define which way is the best to select miRNAs as potential therapeutic molecules while causing the least collateral damage. Here, we summarize a series of variables that must be considered in every pathway for miRNA therapy but also suggest a criterion to define the most biologically significant miRNAs for therapy development: (1) screening for expression profiles, (2) defining interactomes, (3) setting biological relevance, and (4) selecting of master regulators.

As with other therapies, the first step is to understand the overall landscape, and this can be achieved by profiling dysregulated miRNAs. Particularly when proposing combinatory therapy. Traditional methods include performing next-generation sequencing (NGS) or microarrays, and then analyzing the data with edgeR or DeSeq2 bioinformatic pipelines to generate the gene expression profiles [116,117]. A key approach used to determine the miRNA expression profile in drug resistance is to use antimiRNAs or mimics in cells resistant to a certain drug to assess their effect on tumoral cells. In the context to start obtaining this information, a novel approach is the use of biochemical DNA biosensors. These can aid in detecting and identifying miRNAs associated with resistance phenotypes in both clinical and research settings [118]. A more comprehensive yet accurate approach is the implementation of genome editing screening of an upregulated/downregulated profile. Interestingly, CRISPR (clustered regularly interspaced short palindromic repeats) screening has further applications, such as the annotation of novel miRNAs with a role in the disease development or identification of prognostic/diagnostic and monitoring biomarkers [119]. CRISPR screening is used to identify miRNAs that cause resistance to therapy. For instance, miR-3689a-3p causes resistance to sorafenib in HCC [120]. In this sense, miRNA that sensitize cells to therapy can also be identified by CRISPR/Cas9 screening. For example, the *SAGA* (Spt-Ada-Gcn5-acetyltransferase) gene, a transcriptional activator (although not an miRNA) can sensitize AML (acute myeloid leukemia) cells to double-negative T-cell (DNT) therapy by causing DNT-mediated cytotoxicity [121]. In the future, CRISPR screening can be further employed to provide a more comprehensive understanding of this. Furthermore, these applications can greatly benefit from the use of microfluidics chips to isolate and characterize tumor cell heterogeneity at a single-cell level, allowing for the assessment of drug efficacy and resistance [122].

Identifying miRNA targets is the second step in the process of miRNA-based therapy, but validating them is even more important, as is building reliable gene regulatory networks. At the frontline are the well-known bioinformatics tools that predict complementarity in the seed region, such as TargetScan and miRanda [123,124]. Subsequently, more advanced tools incorporate correlation expression and sequencing data, statistical analysis, and even tissue-specific data. Examples are miRTarVis, MIENTURNET (MicroRNA ENrichment TURned NETwork), and miTALOS [125,126,127]. Fewer tools are available that predict targets in a systemic approach, for instance, miRTargetLink2, which uses gene association data [128]. More recently, novel tools have been developed, for instance, agoTRIBE, which is the first tool that predicts miRNA targets in a single-cell context, thus eliminating the need for labor-intensive experiment [129]. Other tools now incorporate machine learning processes such as TEC-mi-Target, which uses a deep-learning image generator of a potential interaction between a miRNA and its plausible target [130]. Remarkably, CRISPR-screening is also a method that has been used for accurate target prediction, by either knockout genes that revert the miRNA-mutant phenotype or by targeting direct interaction sites that drive the tumoral phenotype [131].

Despite the constant implementation of new tools, predicting miRNA targets remains a challenge, especially because it not only depends on mere complementarity of the seed sequence but also because it depends on the cellular context to which an miRNA is subject to regulation. To the interest of this work, when proposing miRNAs as a combinatory therapeutic with TKIs, we are talking about narrowing the pool of targets and thereby making a more feasible therapy. However, the limitation remains as there is not a consensus on which tool integrates the most variables and should therefore be used. It is also worth mentioning the importance of new tools like TEC-mi-Target to study combinatory therapies in which more than one molecule is employed [130]. For instance, molecular docking is widely used in the development of protein-based and small-molecule therapeutics, and it is therefore quite important to set it as a canonical process in RNA-based drug therapy. Several tools are already available to study the interaction of RNA molecules with proteins, mRNAs, and small drugs [132]. As with any other bioinformatics analysis, functional confirmation is required. For this purpose, techniques such as co-precipitation of miRNAs and their interacting molecules are used not only to confirm interaction but also to perform high-throughput screening for initial screening [133].

In regard to cellular function, progress has been made with the development of a new tool called miRSystem, which not only integrates seven algorithms to predict miRNA targets but also incorporates two databases for potential biological functions [134]. Other algorithms integrate miRNA target prediction tools with KEGG (Kyoto Encyclopedia of Genes and Genomes). For instance, VANESA uses the KEGG database to integrate its data with only experimentally validated miRNAs [135].

Although incorporating KEGG data into miRNA networks helps study their functional implications, the KEGG database lacks dynamic cellular changes such as gene expression, the microenvironment- and temporal variations, to name a few. However, these variables can be considered in experimental settings and data analysis of profiling technologies such as NGS and microarrays. Then, what remains unaddressed is how to manage the multimodality capability of regulation that most miRNAs have on hundreds of genes. In this context, miRNAs that converge in regulating a group of genes implicated in the same biological function and/or influence a specific cellular process are known as master regulators, whether they act as oncogenes or tumor suppressors. Identifying master regulators is the last step of the workflow to comprehensively analyze a miRNA as a potential therapeutic or biomarker. Few are the bioinformatic tools available to identify master regulators. For instance, MARINa (master regulator inference algorithm) predicts the transition from one phenotype to another and the maintenance of the latter’s phenotype. This tool has been used to predict the function of transcription factors but has also been integrated with miRNA and mRNA profiling data. On the other hand, VIPER (virtual inference of protein-activity by enriched regulon analysis), based on the same principle as MARINa, and incorporates the regulator’s mode of action, the regulator–target gene interaction confidence of, and the pleiotropic nature of each target gene regulation [136]. A study used these two tools to filter master regulators from a pool of data from the TCGA (The Cancer Genome Atlas). The outcome was the identification of 61 master regulators and 5 potential biomarkers of prognosis for overall survival [137]. To further understand the miRNA network regulation, it is important to consider not only miRNA targets and the downstream effect on gene modulation but also the modulation of miRNAs themselves. DIANA-miRGen v3.0 is a tool that provides cell line-specific miRNA transcription start sites with genome-wide maps of transcription factors (TF) binding sites. TransmiR v2.0 offers a comprehensive database of transcription factors and microRNA interactions based on ChIP-Seq [138].

Besides these bioinformatic pipelines, some studies employ other types of analysis to identify nodes and build networks. For example, Devaraj et al. described a network of 29 microRNA–mRNA with tumor suppressor activity and protective effect, using a bipartite graph theory approach. This study also identified four targets that could be potential therapeutic targets through their inhibition of *VCAN* (versican core protein), *SIL* (SCL-interrupting locus protein), *CD44*, and *MMP14* (matrix metalloproteinase-14) [139]. Furthermore, methods such as deep learning are being used to correlate pre-existing expression data with associated biological functions of miRNA regulatory networks. Joung et al. proposed a probabilistic method to identify highly associated nodes. This approach led them to identify two such nodes, highlighting the importance of this analysis as it not only correlates expression with function but also filters information at a high confidence level [140].

Other well-known tools are available to visualize the results obtained, for instance, Cytoscape (v. 3.10.3) allows the input of three datasets: (1) expression data, (2) miRNA interactions, and (3) pathway enrichment data [141]. FunRich (functional enrichment analysis tool) is an alternative that allows visualization and analysis by biological process, cellular component, molecular function, protein domains, site of expression, biological pathway, transcription factors, and clinical synopsis phenotypic terms.

Despite the availability of a wide range of both functional and bioinformatic tools, there is not a single tool that offers a comprehensive portfolio of analysis necessary to mine the vast data of miRNA networks and their associated biological functions. Furthermore, to our knowledge, there are no tools available to associate specific regulatory networks with miRNAs besides transcription factors. Regarding TKIs, to our knowledge, only one tool is available to analyze miRNA expression in relation to drug response; pharmaco-miR.

## 4. Significance/Conclusions

TKIs have revolutionized targeted therapies for cancer. However, a major challenge for both medical oncologists and patients is resistance, even 6 months after treatment. To overcome resistance, new strategies have been considered, one of which is the use of small molecules such as miRNAs.

The use of miRNAs in combination treatment with TKIs has shown promising results in overcoming resistance to TKIs in various types of cancer, offering great hope (Figure 1). Nevertheless, these studies have not highlighted the difficulties and limitations of using these miRNAs. We highlight the following: (1) delivery method, (2) molecule stability, and (3) immunological response and cytotoxicity.

**Figure 2:**
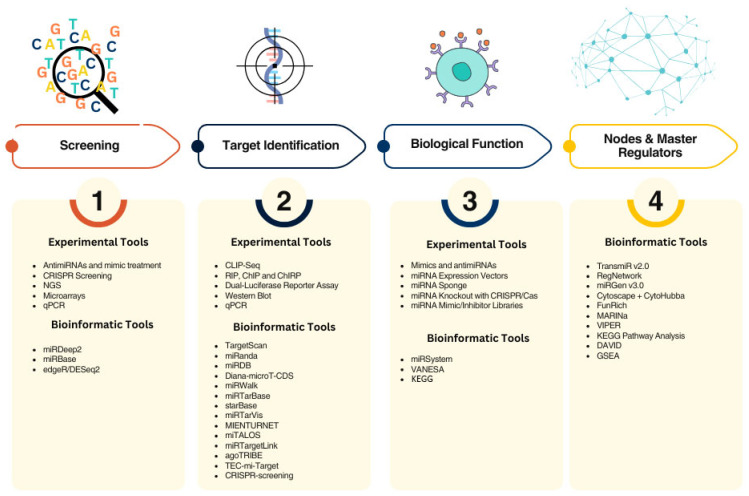
Current experimental and bioinformatic tools in every step of the workflow to select potential microRNA as therapeutics: (1) screening, (2) target identification, (3) biological function, (4) nodes and master regulators.

The primary difficulty, however, is selecting the best miRNA capable of sensitizing a TKI. In this review, we crafted a workflow proposal that includes four steps: (a) screening, (b) target identification, (c) biological function, and (d) nodes and master regulators. Additionally, we propose the use of a series of bioinformatic tools for the development of each step. To our knowledge, this is the first report to outline such a workflow (Figure 2). We hope to provide a pioneering idea that has an impact on breaking the limitations and facilitating the partnership of small molecules (TKIs-miRNAs) to jointly sensitize TKIs in different types of cancer. However, further studies are needed to address and break down other limitations and difficulties.

## Figures and Tables

**Table 1:** Subfamilies of RTKs.

Subfamily	Members
1. EGFR (Epidermal growth factor receptor)	EGFR, ERBB2/3/4 (receptor tyrosine-protein kinase erbB-2/3/4)
2. Ins (Insulin)	INSR (insulin receptor), IGFR (insulin-like growth factor 1 receptor)
3. PDGF (Platelet-derived growth factor)	PDGFRα, PDGFRβ (platelet-derived growth factor receptor alpha/beta), M-CSFR (macrophage colony-stimulating factor receptor), KIT (Mast/stem cell growth factor receptor kit), FLT3L (FMS-like tyrosine kinase 3 ligand)
4. VEGF (Vascular endothelial growth factor)	VEGFR1/2/3 (vascular endothelial growth factor receptor 1/2/3)
5. FGFs (Fibroblast growth factor)	FGFR1/2/3/4 (fibroblast growth factor receptor 1/2/3/4)
6. CCK (colon carcinoma kinase)	CCK4
7. NGF (Beta-nerve growth factor)	TRKA (hig- affinity nerve growth factor receptor), TRKB (BDNF/NT-3 growth factors receptor), TRKC (NT-3 growth factor receptor)
8. HGF (Hepatocyte growth factor)	RON (macrophage-stimulating protein receptor), MET (hepatocyte growth factor receptor)
9. Eph (Ephrin receptors)	EPHA1–6 (ephrin type-A receptor 1–6), EPHB1–6 (ephrin type-B receptor 1–6)
10. AXL (Tyrosine-protein kinase receptor UFO)	AXL, MER (tyrosine-protein kinase mer), TYRO3 (tyrosine-protein kinase receptor TYRO3)
11. TIE (Tyrosine-protein kinase receptor Tie-1)	TIE, TEK (angiopoietin-1 receptor)
12. RYK (Tyrosine-protein kinase RYK)	RYK
13. DDRs (Discoidin Domain Receptor Tyrosine Kinase)	DDR1/2
14. RET (Proto-oncogene tyrosine-protein kinase receptor Ret)	RET
15. ROS (Proto-oncogene tyrosine-protein kinase ROS)	ROS
16. LTK (Leukocyte tyrosine kinase receptor)	ALK (ALK tyrosine kinase receptor), LTK
17. ROR (Inactive tyrosine-protein kinase transmembrane receptor ROR)	ROR1/2
18. MuSK (Muscle, skeletal receptor tyrosine-protein kinase)	MuSK
19. LMR (Lemur tyrosine kinase)	AATYK1/2/3 (serine/threonine-protein kinase LMTK1/2/3)
20. Undetermined	RTK106

**Table 2:** Subfamilies NRTKs.

Subfamily	Members
1. ABL (Tyrosine-protein kinase ABL)	ABL1, ABL2 (ARG)
2. ACK (Activated CDC42 kinase)	TNK1 (non-receptor tyrosine-protein kinase TNK1), TNK2 (ACK1) (tyrosine kinase non-receptor protein 2)
3. CSK (Tyrosine-protein kinase CSK)	CSK, MATK (megakaryocyte-associated tyrosine-protein kinase)
4. FAK (Focal adhesion kinase 1)	PTK2 (FAK), PTK2B (PYK2) (protein-tyrosine kinase 2-beta)
5. FES (Tyrosine-protein kinase Fes/Fps)	FER (tyrosine-protein kinase Fer), FES
6. FRK (Tyrosine-protein kinase FRK)	FRK, PTK6 (BRK) (protein-tyrosine kinase 6), SRMS (tyrosine-protein kinase Srms)
7. JAK (Tyrosine-protein kinase JAK)	JAK1, JAK2, JAK3, TYK2 (non-receptor tyrosine-protein kinase TYK2)
8. SRC-A (Proto-oncogene tyrosine-protein kinase Src)	FGR (tyrosine-protein kinase Fgr), FYN (tyrosine-protein kinase Fyn), SRC, YES1 (tyrosine-protein kinase Yes)
8. SRC-B	BLK (tyrosine-protein kinase Blk), HCK (tyrosine-protein kinase HCK), LCK (tyrosine-protein kinase Lck), LYN (tyrosine-protein kinase Lyn)
9. TEC (Tyrosine-protein kinase Tec)	BTK (tyrosine-protein kinase BTK), ITK (tyrosine-protein kinase ITK/TSK), TEC (tyrosine-protein kinase Tec), TXK (tyrosine-protein kinase TXK)
10. SYK (Tyrosine-protein kinase SYK)	SYK, ZAP70 (tyrosine-protein kinase ZAP-70)

## Data Availability

No new data were created or analyzed in this study. Data sharing is not applicable.
